# Biomarkers of Safety and Immune Protection for Genetically Modified Live Attenuated *Leishmania* Vaccines Against Visceral Leishmaniasis – Discovery and Implications

**DOI:** 10.3389/fimmu.2014.00241

**Published:** 2014-05-23

**Authors:** Sreenivas Gannavaram, Ranadhir Dey, Kumar Avishek, Angamuthu Selvapandiyan, Poonam Salotra, Hira L. Nakhasi

**Affiliations:** ^1^Division of Emerging and Transfusion Transmitted Diseases, Center for Biologics Evaluation and Research, Food and Drug Administration, Bethesda, MD, USA; ^2^National Institute of Pathology, Indian Council of Medical Research, New Delhi, India; ^3^Institute of Molecular Medicine, New Delhi, India

**Keywords:** *Leishmania*, vaccine, genetically modified organisms, live attenuated parasites, vaccine-induced immunity, systems vaccinology, biomarkers of protection

## Abstract

Despite intense efforts there is no safe and efficacious vaccine against visceral leishmaniasis, which is fatal and endemic in many tropical countries. A major shortcoming in the vaccine development against blood-borne parasitic agents such as *Leishmania* is the inadequate predictive power of the early immune responses mounted in the host against the experimental vaccines. Often immune correlates derived from in-bred animal models do not yield immune markers of protection that can be readily extrapolated to humans. The limited efficacy of vaccines based on DNA, subunit, heat killed parasites has led to the realization that acquisition of durable immunity against the protozoan parasites requires a controlled infection with a live attenuated organism. Recent success of irradiated malaria parasites as a vaccine candidate further strengthens this approach to vaccination. We developed several gene deletion mutants in *Leishmania donovani* as potential live attenuated vaccines and reported extensively on the immunogenicity of LdCentrin1 deleted mutant in mice, hamsters, and dogs. Additional limited studies using genetically modified live attenuated *Leishmania* parasites as vaccine candidates have been reported. However, for the live attenuated parasite vaccines, the primary barrier against widespread use remains the absence of clear biomarkers associated with protection and safety. Recent studies in evaluation of vaccines, e.g., influenza and yellow fever vaccines, using systems biology tools demonstrated the power of such strategies in understanding the immunological mechanisms that underpin a protective phenotype. Applying similar tools in isolated human tissues such as PBMCs from healthy individuals infected with live attenuated parasites such as LdCen^−/−^ *in vitro* followed by human microarray hybridization experiments will enable us to understand how early vaccine-induced gene expression profiles and the associated immune responses are coordinately regulated in normal individuals. In addition, comparative analysis of biomarkers in PBMCs from asymptomatic or healed visceral leishmaniasis individuals in response to vaccine candidates including live attenuated parasites may provide clues about determinants of protective immunity and be helpful in shaping the final *Leishmania* vaccine formulation in the clinical trials.

## Introduction

An estimated 100,000 VL cases are reported annually in the endemic foci of northeastern India, Nepal, and Bangladesh alone and ~150 million people are at risk for infection (1). The current programs for elimination of VL include early diagnosis and treatment, coordinated vector control, and effective disease surveillance through passive and active case detection (2). Vector or reservoir control, toxicity of currently available drugs, and increasing parasite resistance underline the need for an effective prophylactic vaccine against leishmaniasis (3).

Estimates of potential economic value of a prophylactic vaccine indicated that even a vaccine with a relatively short duration of protection and modest efficacy could prevent a substantial number of cases at low-cost. Further, a vaccine providing ~5 years duration of protection with as little as 50% efficacy remains cost–effective compared with chemotherapy (4). Development of a prophylactic vaccine against *Leishmania* has gone through a long trajectory that includes phases of systematic selection of antigens, adjuvants, natural immune parameters to identify correlates of protection [reviewed in Ref. (5)]. Despite these advances, there is no effective vaccine even though vaccine is thought to be feasible. Protection against reinfection following a natural infection with *Leishmania major* historically has been the reason for feasibility of a vaccine (6). Similarly people successfully cured from visceral leishmaniasis develop *Leishmania* specific Th1-type cellular-mediated responses and protection against new infections (7). The absence of an effective vaccine to a large extent is related to the absence of clear understanding of correlates of protection. Concerns also remain about the ability of current experimental models to predict protection against natural, sandfly transmitted infection (8). A recent survey of past clinical trials with killed *Leishmania* antigens in Central American countries, Iran, and Sudan showed absence of efficacy against developing CL (3). This further underlines the lack of understanding of the immune mechanisms that drive protection not only in human leishmaniasis but in other parasitic diseases as well. This lack of vaccine efficacy owing to lack of knowledge of correlates of protection is even more powerfully illustrated in the recently concluded malaria vaccine trials where only a subpopulation of the vaccines was protected (9). The parameters measured in the vaccine study did not reveal any correlations that could explain the observed differences. In contrast, tremendous progress has been made in the understanding of vaccine-induced immunity in several viral vaccines including AIDS (10), yellow fever (11), and influenza (12) that showed the power of systematic analysis of early immune responses can have immense value in providing a clear understanding of immune mechanisms of protection.

## Biomarkers of Asymptomatic Carriers of VL-Implications to Vaccine Success

In the Indian subcontinent, humans are the only reservoir of the parasite *L. donovani*. An estimated 100,000 VL cases are reported annually in the endemic foci of northeastern India, Nepal, and Bangladesh, and in addition a significant number of asymptomatic carriers also occur (13). Various epidemiologic studies have reported that asymptomatic infections outnumber clinical VL cases including in India and Nepal (9:1) and Bangladesh (4:1) (14, 15). Thus, early diagnosis and treatment, and effective disease surveillance through passive and active case detection have been identified as key components of *Leishmania* elimination (16). Importantly, the asymptomatic carriers with no overt signs of disease but could potentially develop active VL could serve as reservoirs of parasites in the endemic areas. In practice, more asymptomatic individuals than those with active VL are identified in high endemicity areas (17). A 2-year longitudinal study in the hyperendemic regions in India showed that seropositivity in direct agglutination test (DAT)- and rK39-based tests at baseline cannot predict development of VL (18). Persistence of antibodies over periods of several years against *L. donovani* further complicates the careful identification of active versus past exposure (19). In order to develop biomarkers of parasitemia DAT, ELISA based on rK39 and whole cell lysate and quantitative PCR tests were employed in a recent clinical study (20). Developing tools that can predict progression to VL disease will lead to early intervention and treatment, but also have implications for future vaccination trials. The comparative diagnostic tests revealed the limited complementarity between serology-based tests and DNA-based diagnostic assays underlining the fact that the presence of *L. donovani* DNA is transient, as was also described in a larger survey of asymptomatic children in Brazil (21). Further, the low predicted parasite burden in the asymptomatic carriers in the study (the median 0.1 *L. donovani* DNA equivalents/milliliter of blood) implied that more robust PCR methods need to be developed to detect low parasite burdens. In a whole blood-based IFN-γ release assay in high VL endemic region in India showed that whole blood from active and cured VL cases can produce antigen-specific IFN-γ (19). Secretion of IFN-γ from active VL cases in whole blood assays strongly suggested that no Th1 response deficit exists but the pathogenesis is indicative of immune suppression. The only discriminating factor between active and cured VL was IL-10 where only active VL cases secreted IL-10 (13). This establishes a pattern of biomarkers that will be helpful in a vaccine trial to identify asymptomatic carriers and also raises important considerations for testing of *in vitro* correlates in PBMCs versus whole blood cultures.

## Vaccination Approaches in *Leishmania*

Heat killed *Leishmania* and recombinant antigens have the longest history of clinical trials against CL in parts of South America including Brazil, Ecuador, and also in Iran and Sudan [reviewed in Ref. (3)]. A majority of these trials used *Leishmania* skin test (LST) as a biomarker for vaccine efficacy. This is due to a strong correlation between LST positivity and protection after recovery from the disease caused by several species (22). A retrospective analysis indicated that reproducible evidence of protective efficacy against CL has not emerged from these clinical trials using heat killed *Leishmania* vaccines. Absence of demonstrable efficacy in most of the randomized controlled trials is consistent with the killed whole parasite preparations being inadequate to produce long lasting, relevant immune responses required for protection. Even though vaccinated groups in some trials showed larger LST, the observed immunogenicity was not translated into protective efficacy against CL. Thus measuring LST as a correlate of vaccine-induced immunity has only limited predictive power. However, conversion from negative LST reaction to LST >5 after vaccination has been observed to be associated with significantly lower infection incidence in Brazil, Iran, and Sudan (3). It must be noted that in several of the heat killed *Leishmania* vaccines, BCG was a common adjuvant and the immune reaction caused by BCG compounded the LST-based interpretation significantly. A meta-analysis further confirmed that LST conversion may be associated with an immune response that can provide some protection by its ability to distinguish a subpopulation of responders to leishmanial antigens or BCG after vaccination even though such response had a huge variability (16–68% conversion rate) in these studies (23).

In early vaccination studies, choice of antigens in majority of vaccine formulations related to CL as well as VL was empirical. Systematic studies to identify potential vaccine antigens against VL were undertaken more recently using proteome serology (24), bioinformatics approaches, and reactivity with serum from active VL cases (25, 26). Previous efforts to identify antigens that showed protective efficacy against *L. donovani* infection in experimental VL models include K26/HASPB (27), A2 (28), kinetoplastid membrane protein-11 (29), nucleoside hydrolase (30), cysteine proteinase B (31), and sterol 24-C-methyltransferase (25, 32). Recently, these antigens were evaluated in peripheral blood obtained from a limited number of cases of healed VL and previously unexposed controls to test if the cytokines released in response to *Leishmania* antigen can reveal markers that could predict efficacy of the six candidates (7). If the selected antigens elicit cellular immune responses correlating with protection or cure (e.g., IFN-γ production in previously exposed and cured individuals) that may indicate the potential to be good candidates for prophylactic and therapeutic vaccines. Of the cytokines tested IFN-γ, IL-2, IL-4, IL-5, IL-10, and TNF-α, only soluble *Leishmania* antigen-specific IFN-γ in healed VL cases showed significantly higher secretion compared to controls (7). Interestingly, TNF-α secretion was observed along with high IFN-γ secretion in samples that respond to SLA (7) resembling a pattern of multifunctional Th1 cells that correlate with protection observed against *L. major* infection (33). Interestingly, KMP-11 and K26/HASPB did not show Th1-related IFN-γ production in cured VL subjects even though these antigens were selected on the basis of previous studies with experimental animal models that showed a protective role for KMP-11 and K26/HASPB (27, 29). The observed discrepancy in protective efficacy of these antigens in mouse versus human pre-clinical studies could be due to several reasons including their induction of CD8+ T cells, but not CD4+ cells the latter of which have been shown to be the main source of IFN-γ in cured VL (34) and to limitations in the measurement of responses by CD8+ T cells (7). These studies have demonstrated the importance of comparing antigens for their protective efficacy and allow selection for a future vaccine and importantly revealed the dichotomy in the results obtained in the experimental mouse models and human pre-clinical studies.

Previous studies with HASPB and KMP-11 as prophylactic vaccines indicated good protection associated with the development of CD8+ T cell responses in hamsters and isolated human PBMCs with KMP11 (35, 36) and immunogenicity in dogs against HASPB1 antigen (37). When these antigens were tested in a recent study as potential therapeutic vaccines, experiments with mice revealed that route and dose influenced the breadth and magnitude of the observed CD8+ T cell responses (38). For instance, the response to the HASPB C-terminal epitope was twofold greater in the footpad compared to subcutaneous administration (38). Footpad vaccination induced clear and dose-dependent IgG1 and IgG2a responses, a proxy for CD4+ T cell response but in subcutaneous vaccination such response was undetectable suggesting that route of administration as a determinant of the host response (38). Although significant reduction in splenic parasite burdens was observed, the failure to elicit CD8+ T cell responses indicated that HASPB and KMP11 indicated that they might not be dominant antigens in terms of CD8+ T cell recognition as was seen in several antigen interference studies (39). However, the reduction in the parasite burdens in the immunized mice implied that correlation with strong CD4+ T cell responses upon treatment with *Leishmania* soluble antigen may not be a necessary prerequisite as suggested by the human pre-clinical studies with these antigens. In addition, the data suggested an interesting possibility that for an antigen to be effective as a vaccine candidate, it may not have to be the dominant antigen during natural infection (38).

## Genetically Modified Live Attenuated *L. donovani* as Vaccine Candidates

Though several multi-antigen recombinant protein and DNA vaccines have been and continue to be tested as vaccine candidates, no effective vaccine against VL has been developed so far. In contrast to subunit vaccines, live attenuated parasite vaccines have several advantages in terms of their ability to induce adaptive immune responses relevant to protection by mimicking a natural infection without causing overt disease and likely induce an immune response consistent with protection (40). Early live attenuated *Leishmania* parasites were developed by targeted deletion of dihydrofolate reductase–thymidylate synthase (DHFR-TS) in *L. major* and cysteine proteases cpa and cpb in *L. mexicana* (41, 42). These studies demonstrated the feasibility of using live attenuated parasites for vaccination against CL and provided the rationale for developing and testing several gene deletion mutants in other *Leishmania* species including *L. donovani* subsequently. Further, early progress demonstrated in the vaccination studies in CL using live attenuated parasites led to identification of specific parameters to be evaluated in a discussions sponsored by the WHO (TDR News 2005; http://www.who.int/tdr/publications/documents/tdrnews-issue-75.pdf).

Despite the early progress in CL vaccine studies based on murine models of *L. major* infections, it is well-recognized that the immune mechanisms mediating visceral disease in the liver and spleen caused by *L. donovani* differ significantly from other species of *Leishmania* causing CL and mucocutaneous disease (43, 44). Consequently, vaccine-induced immunity required for protection in CL versus VL is likely to differ in significant ways although cross protection studies indicated common mechanisms of protection (32, 45–49).

Acquisition of protective immunity following leishmanization in cutaneous leishmaniasis (3), development of *Leishmania* specific Th1-type immune response, and protection against new infection in individuals successfully cured from VL (7) and in case of VL a complete *Leishmania* cDNA expression library injected into mice was more protective than subpools of the library plasmids, emphasizing the idea that the whole parasite makes the best vaccine (50). Immunization with live attenuated parasites is likely to deliver several antigens than the limited number possible with subunit or recombinant antigens as revealed by studies in *L. major* (40, 51). Recent success with irradiated *Plasmodium falciparum* sporozoites in inducing strong protection upon intravenous administration further demonstrates the feasibility of using attenuated parasites as vaccine candidates (52). Relative ease of genetic manipulation of genes allowed to produce several gene knock out *L. donovani* parasite strains (5). The live attenuated parasites organisms have the advantage of presenting a complete antigen spectrum like in a natural infection, which may result in a robust immunity unlike subunit vaccines that are limited in antigenic repertoire. The attenuated parasites persist in the host for a limited period of time providing the immune system persistent antigens that allowing for the generation of antigen-specific memory cells that are important for providing a protective response following subsequent infection. Several targeted gene deletions have been carried out to develop *Leishmania*-attenuated vaccine strains. Similar to CL studies, protection against virulent challenge was reported in experimental mouse immunization with *L. donovani* deleted for biopterin transporter (53) A2–rel gene cluster in *L. donovani* (54) SIR2 gene in *L. infantum* (55) and Hsp70-II (56) as immunogens induced protection against virulent challenge in BALB/c mice (Table 1). These experiments demonstrated the potential of generating live attenuated vaccines by targeted gene disruptions. Our laboratory has developed a *L. donovani* mutant (*LdCen*^−/−^) deleted for the centrin gene. Centrin is a growth regulating gene in the protozoan parasites *Leishmania*. *LdCen*^−/−^ is specifically attenuated at the amastigote stage and not as the promastigote (57). The mutant *Leishmania* amastigotes showed cytokinesis arrest in the cell cycle and persisted for a short duration in animals (mice and hamsters) or *ex vivo* in human macrophages and were eventually cleared (48). Mouse immunization experiments revealed that *LdCen*^−/−^ can protect mice against virulent challenge and this protection was accompanied by the induction of robust *Leishmania* specific multifunctional T cell responses (48) as was reported in previous studies that showed strong protection against an *L. major* challenge (33). Immunization experiments also revealed that intrinsic growth defect of *LdCen*^−/−^ amastigotes allows for limited replication in the mouse as was revealed by their clearance after a limited period. Immunization experiments in dogs with *LdCen*^−/−^ revealed that these attenuated parasites can be inducing potent immunogenicity and early indication of protection against virulent challenge (58). Similarly, we developed *L. donovani* mutant (*Ldp27*^−/−^) deleted for the *p27* gene, an essential component of cytochrome *c* oxidase complex involved in oxidative phosphorylation (59). Similar to *LdCen*^−/−^, *Ldp27*^−/−^ parasites also induced *Leishmania*-specific multifunctional T cell responses in mice and showed strong potential as a candidate vaccine (49). We have also developed *L. donovani* mutants deleted for a ubiquitin-like protein in *Leishmania* (*LdUfm1*^−/−^) and the processing enzyme Ufsp (*LdUfsp*^−/−^) that converts the precursor Ufm1 into its conjugatable form (60, 61). *L. donovani* Ufm1 conjugates to and modifies the enzymes involved in β-oxidation of fatty acids. Since the Ufm1 protein is part of a pathway involving activities of several proteins, it is possible to create additional deletion mutants deficient in multiple genes without causing additive loss of virulence thus enhancing the safety of the live attenuated parasites. Together, these examples of genetically altered parasites provide opportunities for testing as live attenuated vaccine candidates in pre-clinical and clinical conditions. Despite the advantages of live attenuated parasites as vaccines, there are considerable challenges. A major concern with live attenuated vaccines is the risk of reversion to a virulent parasite. Hence, biomarkers of safety are essential to assess the genetic and physiological traits of the organism to assess stability of the attenuated parasites in addition to biomarkers of efficacy that are just as important to any kind of anti-*Leishmania* vaccine. Several issues regarding the live attenuated vaccines must be overcome including safety, genetic stability, lack of transmissibility, limited persistence, and conditions of cryopreservation in order for these vaccines to be used in human trials (62). In addition, growing the attenuated organisms in media containing serum of bovine origin constitutes a possible safety risk due to BSE-related hazard. Our recent studies have shown that the live attenuated *L. donovani* parasites can be grown in serum free media (63). The presence of antibiotic markers in the live attenuated organisms can be potential safety concern. Recent clinical trials for treating lung cancer and glioblastoma with tumor cell vaccines or retroviral vectors containing Neo^r^ and Hyg^r^ markers that confer resistance against neomycin and hygromycin suggest that these markers may be permissible in clinical trials (64, 65). However, recent developments in genome engineering methods including use of zinc finger nucleases, transcription activator-like effector nucleases might be pertinent for developing marker free live attenuated *Leishmania* parasites as was the use of Tn5 transposon or thymidine kinase-based antibiotic marker removal (66–69). The ability to create marker free attenuated mutants might also be helpful in creating *Leishmania* parasites lacking multiple genes thus limiting the probability of reversion to virulence due to mutations in secondary loci as observed in lpg2^−/−^ in *L. major* (70). Similarly, infection experiments under immunosuppressive conditions to show limited persistence and sand fly infections post-immunization to show lack of transmissibility might be necessary for demonstrating the safety of live attenuated *Leishmania* parasites. Our preliminary experiments to investigate transmissibility by sand flies have shown that the live attenuated *L. donovani* parasites (*LdCen*^−/−^ and *Ldp27*^−/−^) do not establish an infection in sand flies (Dey et al., unpublished).

**Table 1:** **Genetically modified live attenuated vaccine candidates in *L. donovani* complex**.

Target of deletion	Animal model	Results of immunization	Reference
Biopterin transporter, LdBT1^−/−^	Balb/C	Protective immunity, antigen-specific IFN-γ secretion	Papadopoulou et al. (53)
Centrin 1, LdCen^−/−^	Balb/C mice, hamsters, dogs	Protective immunity against *L. donovani* and *L. braziliensis* challenge. Increased IFNγ, IL-2, and TNF producing cells and IFNγ/IL-10 ratio, presence of multifunctional cells	Selvapandiyan et al. (48), Fiuza et al. (58)
Silent information regulatory two single allele deletion, LiSIR2±	Balb/C	Protective immunity, increased antigen-specific IFNγ/IL-10 ratio	Silvestre et al. (55)
Cytochrome *c* oxidase complex component p27, Ldp27^−/−^	Balb/C	12-Week survival in host, protective immunity against *L. donovani*, *L. braziliensis*, and *L. major* challenge. Increased IFNγ, IL-2, and TNF producing cells and IFNγ/IL-10 ratio	Dey et al. (49)
Heat shock protein 70, LiHsp70^−/−^	Balb/C	Cross protection against *L. major*, high IgG2a relative to IgG1	Carrion et al. (56)

## Immunology of Human VL

The immunopathology of VL has been extensively studied in experimental mouse model, dog model, and to a limited extent in humans. It has long been established that protection against *Leishmania* is derived predominantly from T cell-mediated immunity with both Th1- and Th2-associated cytokines contributing to vaccine-induced resistance (71). Even though the correlates of immunity to human VL are not fully understood, the protection requires antigen-specific CD+4 and CD+8 T cell responses (72). PBMCs from active VL patients typically do not proliferate or produce IFN-γ in response to *Leishmania* antigen where as PBMCs from cured VL cases do proliferate [(73), Avishek et al., unpublished data]. Recent studies have clarified that human VL is not associated with Th2-biased responses dominated by IL-4 and or IL-13 but produce elevated serum IL-1, IL-6, IL-8, IL-12, IL-15, IP-10, IFN-γ, and TNFα (74, 75). The role of IL-10 in promoting pathology of VL has long been demonstrated in human studies. This is primarily accomplished by turning macrophages unresponsive to activation signals. DCs are important for initiating immunity and further modifying the immune response. Secretion of cytokines by DCs strongly influences the outcome of T cell responses. Stimulation of DCs to produce IL-12 drives creation of antigen-specific IFN-γ secreting Th1-type CD4 and CD8 cells that aid in resolution of infection (76). Several human studies indicated that IFN-γ secretion is the biomarker for protection even though both VL and CL can progress despite the presence of a Th1-type of response. IL-10 expression is the only other marker that has been consistently shown to negatively correlate with the protection. Elevated serum levels of IL-10, IL-6, and IL-8 in serum have been shown to be associated with active disease (73).

Poor understanding of the mechanism of immune protection has thus impaired development of effective vaccine against human VL so far. This is in many respects paralleled by attempts at developing a vaccine against malaria. More recently, results from the clinical trials with RTS,S/AS01 in the infants have underlined the importance of biomarkers of efficacy. Efficacy of RTS,S/AS01 was only 26% against sever malaria (9). Absence of knowledge about the nature of adaptive immune responses in certain protected population in malaria endemic areas and whether such immunological correlates of protection can be mimicked by vaccination still remain unanswered. The protection observed only in small fraction of infants suggests that it is important to determine which vaccine elicited responses are necessary for such protection (77). Since typical clinical trials can last for several years, absence of knowledge on biomarkers of protection can considerably impede the progress of vaccine development. This is reflected by the limited success rate (22%) observed in vaccine development (78). Comprehensive analysis of antibodies, cytokines, immune cells, and whole-genome transcription to identify key host responses associated with an effective protection would be necessary to reveal those answers.

## Systems Biological Approaches in Vaccine-Induced Immunity

Majority of the currently licensed vaccines have mostly developed empirically, and protection by these vaccines is generally conferred by antigen-specific antibodies, which prevent infection. Many viral vaccines, such as the live attenuated vaccines, work by mimicking pathogens, to stimulate lasting and protective immunity in the host (79). Only recently, the immune mechanisms underlying the protection are beginning to be understood in the otherwise empirically developed viral vaccines. Recent technological advances in molecular genetics, molecular and cellular immunology, structural biology, bioinformatics, computational biology, nanotechnology, formulation technologies, and systems biology have facilitated new developments in antigen discovery/design, adjuvant discovery, and immune monitoring that offer substantial potential for discovering new biomarkers of protective immunity, and identify the limitations of animal models for screening and prioritizing human vaccines (80). Recent progress in understanding how the innate immune system recognizes microbial stimuli and regulates adaptive immunity is being applied to vaccine discovery in what is termed “systems vaccinology” (81). Systems vaccinology is an offshoot of systems biology for which tools of a number of high-throughput technologies including DNA microarrays, RNA-seq, protein arrays, deep sequencing, mass spectrometry along with sophisticated computational tools have been originally developed (81). Together, these tools enable system-wide unbiased molecular measurements, which can then be used to reconstruct the perturbations in the immune networks (82).

The tools of systems biology were first applied for the highly effective yellow fever vaccine YF-17D, which is a live attenuated virus. Yellow fever vaccine has been one of the most successful vaccines known. Past immunological studies have revealed that YF-17D induces neutralizing antibodies, cytotoxic T cells and Th1/Th2 cells, and signal via Toll-like receptors 2, 7, 8, and 9 on subsets of dendritic cells (83). Querec et al. (11) and Gaucher et al. (84) first applied systems biological approaches in evaluating immune responses to vaccination to discern new and fundamental insights about the workings of the immune system by detailed study of innate and adaptive immune responses to vaccination (Yellow Fever Vaccine) in humans. When the PBMCs from the vaccinated and control groups were tested in human microarray hybridization experiments, results revealed that genes of the antiviral type I interferon pathway, complement pathway, and inflammasome were induced 3–7 days after vaccination, concomitant with the development of the adaptive immune response. In addition, a broad spectrum of gene regulations for innate sensing, including TLR7, RIG-I, MDA5, and LGP2, and for innate signaling, including JUN, STAT1, IRF7, and RNF36, was also observed. Analysis of immune networks revealed that it is possible to predict titers of neutralizing antibodies by the early gene expression signatures.

Later studies with seasonal influenza model using systems biology approaches compared immune responses to a live attenuated virus vaccine, or a trivalent-inactivated vaccine (12). This comparison revealed important differences between the live attenuated versus trivalent-inactivated vaccines. The live attenuated vaccine induced the expression of several interferon-related genes, common to live viral vaccines where as, the inactivated vaccine, induced genes highly expressed in plasma B cells. Such comparative analysis of gene expression in human microarrays in the first week of vaccination allowed prediction of antibody responses in the vaccines based on the expression of markers such as the receptor gene for B cell growth factor BLyS (12). This clearly demonstrated that analysis of early transcriptomic profiles from the peripheral blood can be used in identifying novel immune networks and also predict vaccine efficacy.

The gene expression profiles once obtained can also be utilized to identify if the correlates of protection are universally applicable. For instance, the metabolic changes of the aging body, including the increased presence of apoptotic cells and of oxidative stress induce the immune system to change its “quiescent” state to a different, often higher level of basal activation [reviewed in Ref. (85)]. Similarly, the role of sex hormones in affecting the vaccine-induced immunity is not often studied. A meta-analysis of the yellow fever vaccine study revealed substantially higher expression signatures in the toll-like receptor–interferon signaling in women compared to men (86). Similar studies with influenza vaccine revealed an immunosuppressive role for testosterone in influenza vaccination. Unexpectedly, this immunosuppression was linked to genes that participate in lipid metabolism including leukotriene A4 hydrolase, revealing the power of systems analysis (87). The main appeal of using systems biology approaches to examine vaccine-induced immunity is the ability to study early responses in peripheral blood by comparing genes and pathways induced before/after immunization. This paradigm can be applied to compare efficacy of different vaccine formulations including live attenuated parasite vaccines, recombinant antigen vaccines if knowledge of biomarkers of protection is available.

Since immune cells migrate through the blood stream between lymph nodes, spleen, and peripheral tissues obtaining meaningful information of an immune response from this dynamic mix of cells remains a complex challenge. Several attempts have been made at decomposing the gene expression profile from a heterogeneous mix of cells into that of respective cell types based on unique gene expression profiles independently obtained from a pure cell population such as monocytes, lymphocytes, neutrophils, eosinophils, and basophils (88).

With the development of genomic and proteomic tools along with advances in computational approaches, global profiling of cellular states in terms of gene and protein expression has been applied to study a broad range of immunological phenomena. These approaches have been used to study T cell activation signatures (89, 90), blood cell states in patients with autoimmunity (91), and the responses of host cells to HCV infection (92).

Even though the approaches outlined here can provide fundamental insights into the immune responses to complex pathogens, it must be noted that so far only viral vaccines were studied. In most such cases, protection is mediated by antibodies whereas clearance of parasitic pathogens needs predominantly cell-mediated immunity. The role of antibody responses in protection is at best inconclusive in human leishmaniasis (73). In addition, it is conceivable that parasites engage with human immunity in multiple levels as is evidenced by immune suppression in humans with active VL. Computational approaches developed for the analysis of innate immunity point to the possibility of such analysis in human VL (93).

## Conclusion

A major shortcoming in the vaccine development is the inadequate predictive power of the early immune responses mounted in the host against the vaccines. Also, for the live attenuated parasite vaccines, the primary barrier against widespread use remains safe in terms of avirulence of the parasites in host. Therefore, understanding of the pathogenesis of live attenuated parasites such as *LdCen*^−/−^ in human PBMC in different clinical groups will provide valuable information regarding avirulence of these parasites and efficacy in terms of immune- and non-immune-related responses prior to the evaluation in human trials. Recent influenza vaccine studies substantiated that the role of non-immune parameters in protective immunity is generally under appreciated and often missed in conventional vaccine studies that measure only immunological parameters. Obtaining such information via systems biology approaches, as has been applied to study viral vaccines, will enable us to understand how vaccine-induced responses are coordinately regulated in healthy, asymptomatic infected individuals, and individuals recovered from VL. This will provide information regarding correlates of protection as well as biomarkers of safety and enable identification of immune and non-immune predictors hitherto unidentified to *Leishmania* antigens or live attenuated vaccine candidates in the human cells and might be useful in shaping the final vaccine formulation in the clinical trials. Studies comparing expression profiles of PBMCs from bonafide asymptomatic carriers with those that acquired protective immunity following clinical cure will likely provide biomarkers other than the IFN-γ secretion as is currently practiced. Understanding of immune modulators that confer protection in VL can lead to new targets for immune therapy.

## Conflict of Interest Statement

The authors declare that the research was conducted in the absence of any commercial or financial relationships that could be construed as a potential conflict of interest.
